# Regional diffuse fibrosis and strain characteristics differ between children with hypertrophic and dilated cardiomyopathy

**DOI:** 10.1186/1532-429X-17-S1-P287

**Published:** 2015-02-03

**Authors:** Jyoti K Patel, Kevin K Whitehead, Matthew A Harris, Marc S Keller, Christopher M Kramer, Frederick H Epstein, Kimberly Y Lin, Joseph W Rossano, Mark A Fogel

**Affiliations:** Pediatrics, Children’s Hospital of Philadelphia, Philadelphia, PA USA; Radiology, Children’s Hospital of Philadelphia, Philadelphia, PA USA; Radiology and Medicine, University of Virginia Health System, Charlottesville, VA USA; Pediatrics and Radiology, Children’s Hospital of Philadelphia, Philadelphia, PA USA; Biomedical Engineering, University of Virginia Health System, Charlottesville, VA USA

## Background

Diffuse fibrosis and strain are abnormal in adult cardiomyopathy patients and in children exposed to anthracyclines, but have not been well studied in pediatric hypertrophic (HCM) and dilated (DCM) cardiomyopathy. We hypothesized that HCM and DCM patients have different diffuse fibrosis and strain characteristics.

## Methods

Consecutive pediatric subjects with HCM (n=12) or DCM (n=8) undergoing routine clinical CMR were recruited. Native and post contrast T1 mapping was performed using Modified Look-Locker inversion recovery (MOLLI) sequences in all subjects. In 10 subjects, the maximum and mean circumferential strains were also examined using Displacement Encoding With Stimulated Echoes (DENSE). Data for both T1 mapping and strain were evaluated in a 16 segment cardiac model.

## Results

Study subjects (M16, F4) had an average age at MRI of 13.5±4.1 years with no difference in HCM or DCM age. Subjects with HCM had significantly lower partition coefficients (0.45±0.09 vs 0.48±0.10, p=.01) and higher post T1 values (452±58 vs 430±59 msec, p=.001) than those with DCM. DCM subjects had lower maximal strain (-12.5±4.8 vs -15.7±5.8, p=.001) and mean strain (-4.4±3.6 vs -6.7±3.8, p=.0004). Native T1 values had a positive correlation with LV mass in HCM subjects (R=.57, p =.05), suggesting increased fibrosis in subjects with more significant hypertrophy. There was no correlation between T1 values and regional myocardial strain, left ventricular ejection fraction, or the presence in the subject of late gadolinium enhancement. In the total cohort, there was a significantly different partition coefficient for each heart slice (base 0.43±0.07, mid 0.52±0.09, apex 0.44±0.1; p=.006).

## Conclusions

There was a significant difference in the T1 values and partition coefficients in pediatric subjects with HCM and DCM, suggesting relatively increased fibrosis in HCM. This also correlated to severity of disease in HCM as assessed by LV mass. Additionally, DCM subjects have an impaired strain pattern compared to HCM subjects. Further, there is a dissociation between regional diffuse fibrosis and strain in cardiomyopathy patients. The difference in partition coefficients through the left ventricular long axis suggests that the disease processes are not uniform throughout the myocardium.

